# Sources of personal PM_2.5_ exposure during pregnancy in the MADRES cohort

**DOI:** 10.1038/s41370-024-00648-z

**Published:** 2024-02-07

**Authors:** Yan Xu, Karl O’Sharkey, Jane Cabison, Marisela Rosales, Thomas Chavez, Mark Johnson, Tingyu Yang, Seung-Hyun Cho, Ryan Chartier, Brendan Grubbs, Nathana Lurvey, Deborah Lerner, Frederick Lurmann, Shohreh Farzan, Theresa M. Bastain, Carrie Breton, John P. Wilson, Rima Habre

**Affiliations:** 1 Spatial Sciences Institute, University of Southern California, Los Angeles, CA USA; 2 Department of Population and Public Health Sciences, University of Southern California, Los Angeles, CA USA; 3 RTI International, Research Triangle Park, NC USA; 4 Department of Obstetrics and Gynecology, University of Southern California, Los Angeles, CA USA; 5 Eisner Health, Los Angeles, CA USA; 6 Sonoma Technology, Inc., Petaluma, CA USA; 7 Department of Civil & Environmental Engineering, Computer Science, and Sociology, University of Southern California, Los Angeles, CA USA

**Keywords:** Personal exposure, PM_2.5_, Source apportionment, pregnancy, Secondhand smoking source, Traffic source

## Abstract

**Background:**

Personal exposure to fine particulate matter (PM_2.5_) is impacted by different sources each with different chemical composition. Determining these sources is important for reducing personal exposure and its health risks especially during pregnancy.

**Objective:**

Identify main sources and their contributions to the personal PM_2.5_ exposure in 213 women in the 3rd trimester of pregnancy in Los Angeles, CA.

**Methods:**

We measured 48-hr integrated personal PM_2.5_ exposure and analyzed filters for PM_2.5_ mass, elemental composition, and optical carbon fractions. We used the EPA Positive Matrix Factorization (PMF) model to resolve and quantify the major sources of personal PM_2.5_ exposure. We then investigated bivariate relationships between sources, time-activity patterns, and environmental exposures in activity spaces and residential neighborhoods to further understand sources.

**Results:**

Mean personal PM_2.5_ mass concentration was 22.3 (SD = 16.6) μg/m^3^. Twenty-five species and PM_2.5_ mass were used in PMF with a final *R*^2^ of 0.48. We identified six sources (with major species in profiles and % contribution to PM_2.5_ mass) as follows: secondhand smoking (SHS) (brown carbon, environmental tobacco smoke; 65.3%), fuel oil (nickel, vanadium; 11.7%), crustal (aluminum, calcium, silicon; 11.5%), fresh sea salt (sodium, chlorine; 4.7%), aged sea salt (sodium, magnesium, sulfur; 4.3%), and traffic (black carbon, zinc; 2.6%). SHS was significantly greater in apartments compared to houses. Crustal source was correlated with more occupants in the household. Aged sea salt increased with temperature and outdoor ozone, while fresh sea salt was highest on days with westerly winds from the Pacific Ocean. Traffic was positively correlated with ambient NO_2_ and traffic-related NO_*x*_ at residence. Overall, 76.8% of personal PM_2.5_ mass came from indoor or personal compared to outdoor sources.

**Impact:**

We conducted source apportionment of personal PM_2.5_ samples in pregnancy in Los Angeles, CA. Among identified sources, secondhand smoking contributed the most to the personal exposure. In addition, traffic, crustal, fuel oil, fresh and aged sea salt sources were also identified as main sources. Traffic sources contained markers of combustion and non-exhaust wear emissions. Crustal source was correlated with more occupants in the household. Aged sea salt source increased with temperature and outdoor ozone and fresh sea salt source was highest on days with westerly winds from the Pacific Ocean.

## Introduction

Personal exposure to particulate matter with aerodynamic diameter <2.5 µm (PM_2.5_) is impacted by indoor, outdoor, and personal activity-related (i.e., behaviors) sources in various microenvironments, spaces, and neighborhoods that individuals typically encounter [1, 2]. Prenatal exposure to PM_2.5_ specifically is associated with adverse maternal and fetal health outcomes [3–5]. Exposure in the 3rd trimester of pregnancy, when most fetal weight gain occurs is thought to be associated with low birth weight and impaired growth [6–8]. The toxicity of PM_2.5_ and its subsequent impact on health is driven by its chemical composition and main sources contributing to it [8–10]. Identifying and quantifying the main sources of personal PM_2.5_ exposure can shed light on particular mixtures that might pose a greater health risk and might otherwise be missed by solely investigating total PM_2.5_ mass concentration as a whole. This is particularly important in environmental health disparities contexts and for specific vulnerable populations such as pregnant women for whom meaningful recommendations to reduce exposures and health risks are needed [11, 12].

Main sources of PM exposure are generally resolved using source- and receptor-oriented modeling approaches [13, 14]. Based on the mass balance principle [15], receptor-oriented approaches utilize speciated measurements at receptors (i.e., receiving) to identify and quantify major sources (or source groups) impacting that location or individual [16, 17]. One of the most commonly used receptor models is the Positive Matrix Factorization Model (PMF) which solves for sources and their chemical profiles without pre-assuming what they are [18–20].

Several studies have conducted source apportionment in outdoor and indoor environments and investigated their health impacts [3, 21, 22]. For example, vehicular emissions, wood smoke, natural gas combustion, ship emissions, secondary aerosols, fresh and aged sea salt, and soil/road dust sources were resolved in outdoor PM (various size ranges) in Los Angeles, CA communities [23, 24]. Hasheminassab et al. [25] found mobile sources were the major contributor to both indoor (39 ± 21%) and outdoor (46 ± 17%) PM_2.5_ mass in three retirement homes in Los Angeles, Habre et al. [26] found cooking, cleaning, candle/incense burning, and smoking contributed significantly to indoor PM_2.5_ concentrations in New York City residences.

However, personal exposure to PM_2.5_ and its sources can be more complex to discern for several reasons. First, while personal monitoring (sampling air in the personal breathing zone) is considered the gold standard approach in external individual-level exposure assessment [27, 28], the cost and burden of conducting these studies is still high especially during pregnancy [29, 30]. Second, individuals get exposed to PM_2.5_ in multiple microenvironments and locations, often in close proximity to indoor sources or while mobile, sometimes generating PM_2.5_ from their activities (e.g., burning candles), and are always impacted by outdoor air pollution that infiltrates indoors or into the personal breathing zone [27, 28, 31, 32]. As such, disentangling sources that contribute to personal exposure can be more challenging compared to outdoor or indoor studies. This challenge is reflected by the relatively small number of studies conducting source apportionment in personal samples, most of which are collected 12 to 48 hour integrated PM samples [2, 33–35].

Of these, Özkaynak et al. [35] found that personal PM_10_ exposure was much higher than outdoor and indoor PM_10_ concentrations in 178 nonsmoking residents in Riverside, CA, and that these explained 16% and 50% of the variation in personal exposures, respectively. Cooking and smoking were important sources of personal exposure. Minguillón et al. [34] found wide variation in personal PM_2.5_ exposures of 54 pregnant women and reported on limitations of questionnaire data (e.g., recall error, accuracy of time and location of travel and activities) in helping to resolve sources.

To the best of our knowledge, no studies to date have conducted source apportionment on personal PM_2.5_ exposure samples in the 3rd trimester of pregnancy. We aimed to chemically characterize the composition of personal PM_2.5_ and resolve its main sources in the MADRES (Maternal And Developmental Risks from Environmental and Social Stressors) cohort in Los Angeles, CA. To accomplish this goal, we analyzed filter-based data from a personal monitoring sub-study in MADRES using the United States Environmental Protection Agency (EPA) PMF model [19]. We leveraged questionnaire data, geolocation monitoring (GPS), and geospatial modeling of environmental exposures (in residential neighborhoods and activity spaces) to confirm predicted source identities and understand how their mass contributions vary in relation to personal behaviors, indoor/outdoor sources, and time-activity patterns.

## Methods

### Study design

A total of 213 women in their 3rd trimester enrolled in the larger MADRES cohort were recruited into this personal monitoring sub-study between October 2016 and March 2020. MADRES is an ongoing prospective pregnancy cohort focused on predominantly low-income, Hispanic women and their babies residing in Los Angeles, CA. MADRES aims to address critical gaps in understanding environmental health disparities and the impacts of air pollution and social stressors on maternal and child health. The details of eligibility, enrollment, and follow-up of participants are described elsewhere [36]. Briefly, eligible participants for this sub-study were in the 3rd trimester at the time of recruitment, ≥18 years of age, and could speak either English or Spanish fluently. In the initial design, people living in a household with an active smoker were excluded to reduce the impact from smoking on personal PM_2.5_ exposures. However, in order to encourage all participants to contribute to sub studies, the non-smoking household criterion was not applied consistently throughout the study and was eliminated by the end of 2018. Informed consent was obtained for each participant. The University of Southern California’s Institutional Review Board (IRB) approved the study protocol.

### Data collection

The 48-hr integrated personal PM_2.5_ measurements were collected to characterize the composition of personal PM_2.5_ and identify its main sources in the MADRES cohort through source apportionment analysis. Several other data sources were used from MADRES questionnaires and measurements and from external data sources in a second follow-on bivariate analysis to confirm source identities and understand the personal drivers that affect the mass contribution of each source. These included the following: questionnaires (collected at trimester 1, 2, and 3, respectively), GPS-derived time-activity patterns and environmental exposures within activity spaces from the personal monitoring study, modeled residential environmental exposures, and outdoor EPA PM_2.5_ chemical speciation data from a central site.

#### Personal PM_2.5_ measurements

The personal PM_2.5_ sampling design and protocol in the 3rd trimester is described in detail in O’Sharkey et al. [37] and Xu et al. [38]. Briefly, personal, 48-h integrated PM_2.5_ measurements were collected using a Gilian Plus Datalogging Pump (Sensidyne, Inc.) operating on a 50% cycle at 1.8 lpm flow rate with the sampling inlet located in the breathing zone. The pump is connected to a PM_2.5_ Harvard Personal Environmental Monitor (PEM) size-selective impactor with a 37 mm Teflon filter (2 µm pore size; Pall, Inc.). Participants were asked to wear the sampling device for the entire data collection period with a few exceptions. These included when it is unsafe to do so (e.g., driving), showering, or sleeping, in which case they were instructed to place the device near them in an unobstructed location.

Filters were analyzed gravimetrically to determine PM_2.5_ mass using an MT5 microbalance (Mettler Toledo, Columbus, OH, USA) in a dedicated humidity- and temperature-controlled chamber at the USC Exposure Analytics Laboratory. Filters were then sent to RTI International (Research Triangle Park, NC) to determine elemental composition of the following 33 species using Energy Dispersive X-Ray Fluorescence (EDXRF): barium (Ba), calcium (Ca), chlorine (Cl), copper (Cu), iron (Fe), potassium (K), magnesium (Mg), manganese (Mn), sodium (Na), nickel (Ni), sulfur (S), silicon (Si), titanium (Ti), zinc (Zn), aluminum (Al), bromine (Br), cobalt (Co), phosphorus (P), lead (Pb), selenium (Se), strontium (Sr), vanadium (V), cesium (Cs), zirconium (Zr), chromium (Cr), rubidium (Rb), arsenic (As), indium (In), silver (Ag), antimony (Sb), tin (Sn), cerium (Ce), and cadmium (Cd). Filters were also analyzed for concentrations of black carbon (BC), brown carbon (BrC), and environmental tobacco smoke (ETS) using a seven-wavelength optical transmittance integrating sphere method [39, 40].

#### Questionnaires

Participants completed interviewer-administered questionnaires in trimester-specific visits as part of the larger MADRES cohort and an exit survey after completing the 48-hr monitoring period as part of the personal monitoring sub-study (Table S1 and S2). Data obtained from the MADRES questionnaires include the following: demographics (e.g., age, race, education, employment, income), housing characteristics (e.g., type of dwelling, building age). In addition, data on the following were available from the exit survey: time-activity patterns (e.g., time spent indoors and outdoors, commuting), home ventilation (e.g., window open, air conditioner use), current tobacco smoke exposure (primary and secondhand), and presence of any significant indoor sources of PM_2.5_ such as cooking or candle burning [36]. Participants’ home addresses at the 3rd trimester study timepoint were geocoded for residential exposure assessment.

#### Residential environmental exposure assessment

Daily ambient concentrations of PM_2.5_, PM_10_, nitrogen dioxide (NO_2_), and ozone (O_3_) were interpolated at the residence using inverse distance squared weighted interpolation from US EPA Air Quality System data [36]. Daily local traffic-related nitrogen oxides (NO_*x*_) concentrations at the residence were estimated using the CALINE4 line source dispersion model by roadway class [41]. Daily meteorology (temperature, precipitation, specific humidity, relative humidity, downward shortwave radiance, wind direction and wind speed) was assigned based on a 4 km × 4 km gridded model developed by Abatzoglou [42]. Forty-eight-hour integrated averages were calculated from daily measurements to correspond to the personal monitoring dates. For wind direction, four categories were created based on the 48-hr mean (arithmetic mean of two vector averages) as follows: 0–90° as wind blowing from northeast (NE), 91–180° as southeast (SE), 181–270° as southwest (SW), and 271–360° as northwest (NW), where a direction of 0° is due North on a compass.

#### GPS-derived time-activity patterns and environmental exposures within activity spaces

Smartphones with the study-developed madresGPS Android app pre-installed and programmed were used to log participants’ geolocation (GPS and metadata) and motion sensor data continuously at 10-s intervals for the 48-hr monitoring period. Data were then analyzed to derive time activity patterns as minutes spent staying at home or non-home locations (assumed to be indoors) and minutes spent on the road (or in transit, travel mode unknown) and then converted to percentages out of the 48-hr period for use in the analysis. The methods used to derive time-activity patterns were based on [43, 44] and described in more detail in Xu et al. [38].

GPS data was also used to construct 48-hr activity spaces and calculate environmental exposures encountered within them [38]. Briefly, activity spaces are defined as the local areas that individuals interact with when they move around during their daily activities [45, 46]. We constructed activity spaces using Kernel Density Estimation (KDE) method for each individual and then calculated the following environmental exposures within them: walkability index score, Normalized Difference Vegetation Index (NDVI, greenness), parks and open spaces, traffic volume on primary roads, and road lengths (for primary and secondary roads combined and for minor streets). Data sources for these measures are listed in Table S3. Briefly, KDE integrates time and space to account for durations of time spent at certain locations and incorporates a distance decay kernel function to assign higher weight to environmental features closer to the locations where participants spent the most time in (compared to locations they passed through) using pre-defined bin (e.g., 25 m) and neighborhood sizes (e.g., 250 m) [45, 47]. Activity space calculations were conducted in ArcGIS Pro 2.5 (Esri, Redlands, CA).

#### Outdoor PM_2.5_ chemical speciation in study region

PM_2.5_ metals and carbonaceous components concentrations were available every third day from the Chemical Speciation Network (CSN) [48] at the Downtown Los Angeles monitoring station—the most proximal and central site in the study area—and were downloaded from the EPA Air Quality System. Then, the data was linked to personal monitoring 48- hr time periods based on the overlapping dates.

### Data analysis

Descriptive statistics were first conducted to plot the distributions of all the variables (e.g., individual/residential environment data) for the participants. Then, using USEPA PMF5.0 model, source apportionment analysis was performed on personal PM_2.5_ measurements to identify main sources, along with their contributions to PM_2.5_ mass. Once sources were resolved, bivariate analysis was conducted between each PM_2.5_ source and several variables describing personal behaviors, indoor/outdoor sources, and time-activity patterns to confirm source identities and understand the personal drivers that affect PM_2.5_ exposures.

#### Descriptive analysis

Descriptive statistics were calculated in SAS 9.4 (SAS Institute Inc) to describe the distribution of personal PM_2.5_ mass concentration, elemental components and carbon fractions (used in final PMF analysis), questionnaire variables (home ventilation, time-activity patterns, indoor sources, etc.), and environmental exposures (residential or GPS-derived).

#### Source apportionment analysis

The USEPA PMF 5.0 model was used to resolve and identify major sources of PM_2.5_ and quantify their mass contributions using measured concentrations and sample-specific uncertainties as inputs. Briefly, the PMF model uses factor analysis to identify source contributions and profiles for a given number of sources through solving the following equation: [19, 20, 49]

1 $${X}_{{ij}}=\mathop{\sum }\limits_{k=1}^{n}{g}_{{ik}}{f}_{{kj}}+{e}_{{ij}}$$

where *X*_*ij*_ represents the concentration of chemical component *j* in sample *i*, *g*_*ik*_ represents the mass contribution of factor *k* in sample *i*, *f*_*kj*_ represents the loading of chemical component *j* on factor *k*, and *e*_*ij*_ is the residual error for sample *i* and species *j*.

The PMF model solves Eq. (1) by minimizing the sum of squares object function *Q* for a given number of factors *k*: [20, 49]

2 $$Q=\mathop{\sum }\limits_{i = 1}^{n}\mathop{\sum }\limits_{j =1 }^{m}\left[{\frac{{e}_{{ij}}}{{u}_{{ij}}}}\right]^{2}$$

where *u*_*ij*_ is the uncertainty of species *j* in sample *i*. The model decomposes the concentrations matrix into a contributions *g* matrix and profiles *f* matrix and constrains results to be positive (or not significantly negative) [20, 50]. Each observation is individually weighted by its uncertainty in Eq. (2); therefore, samples with higher analytical uncertainties will have less influence on the solution.

Based on the PMF-calculated signal-to-noise ratio (S/N) indicating the degree of analytical noise relative to the concentration of each species [19], we categorized species as “Bad” (S/N ≤ 0.2, excluded from analysis), “Weak” (0.2 < S/N < 1, downweighted in the analysis), and “Strong” (S/N ≥ 1, retained). Although Pb and V had S/N < 0.2, they were included in the analysis as potentially important tracers of traffic and fuel oil, respectively, and set to “Weak.”

Of the 36 elemental and carbon species, the following 16 were finally included as “Strong” in the PMF analysis: BC, BrC, Ba, Ca, Cl, Cu, Fe, K, Mg, Mn, Na, Ni, S, Si, Ti, and Zn. We also included 9 “Weak” species as follows: Al, Br, Co, ETS, P, Pb, Se, Sr, and V. PM_2.5_ mass was designated as the total variable. An extra 10% modeling uncertainty was added to account for sampling or modeling errors not captured in the sample-specific analytical uncertainties [19]. Missing concentrations were replaced by species’ medians. One out of 213 (0.5%) samples were excluded as outliers based on multiple species’ concentrations.

Solutions with five to seven factors were scanned first to decide upon a reasonable factor number in a final model with 100 runs. *Q* values were checked for no undue influence from outliers and no local minimum solutions. Based on chemical loading profiles and prior knowledge, the optimal number of sources was selected which provided the most physically interpretable solution [50]. The convergent solution with the lowest *Q*_*robust*_ value (goodness-of-fit parameter excluding points with uncertainty-scaled residuals greater than 4) was selected [19]. Fpeak rotations, where positive *F* peak values sharpen the *F* matrix and negative values sharpen the *G* matrix were performed next to refine the solution. The optimal Fpeak value for solution rotation was chosen based on the smallest change in *Q* [19]. Residuals and *R*^2^’s for each species were checked for normality and model fit, respectively. Finally, diagnostics analysis of Displacement (DISP), Bootstrap (BS) (100 bootstraps, 0.6 minimum correlation), and Bootstrap-Displacement (BS-DISP) were performed to estimate the variability in the PMF solution under different scenarios. DISP focuses on effects of rotational ambiguity in the profiles or loadings; BS identifies whether there are a small set of observations that can disproportionately influence the solution; and BS-DISP includes effects of random errors and rotational ambiguity [19].

#### Bivariate confirmatory analysis of source identities and drivers

Bivariate analyses were conducted to further confirm source identities and examine trends in their mass contributions. Scatterplots, boxplots, and nonparametric statistics (Spearman correlations for continuous variables and Kruskal-Wallis test for categorical ones) were used to describe and test the relationships of select factors (selected from the literature and prior knowledge) with each source’s predicted mass contributions. These included demographics, time-activity patterns, home characteristics, indoor air pollution sources, and outdoor residential and activity space based environmental exposures as described earlier. Categorical variables with unbalanced values (≥85% of the records have one value) or with high missingness (≥80%) were dropped from further analysis.

## Results

### Descriptive statistics

Most of the participants (>98%) resided in central and east Los Angeles, CA. The majority were Hispanic (78%), working (48%) during the 3rd trimester, and completed grade 12 or less (55%) (Table S1). Mean age was 28 years at consent (range 18–45 years) and mean parity was 2 (range 1–6). Among participants who reported annual household income, most of them had annual household income <$30,000 (68%).

Personal PM_2.5_ mass and component concentrations are provided in Table 1. Mean (SD) PM_2.5_ concentration was 22.3 (16.6) μg/m^3^. The optical carbon fractions BC, BrC, and ETS combined constituted on average 17% (3.8) μg/m^3^ of the total PM_2.5_ mass.

**Table 1:** PM_2.5_ mass and chemical component concentrations (in units of ng/m^3^ unless otherwise noted).

	*N*	Mean	SD
PM_2.5_ mass (μg/m^3^)	209	22.3	16.6
Carbon species			
BC (μg/m^3^)	209	1.1	1.7
BrC (μg/m^3^)	206	1.1	0.8
ETS (μg/m^3^)	210	1.6	6.1
Elements			
Al	212	12.4	47.2
Ba	212	14.2	13.5
Br	212	3.0	3.1
Ca	212	85.6	141.2
Cl	212	126.5	253.2
Co	212	0.5	0.8
Cu	212	18.7	12.2
Fe	212	122.2	110.7
K	212	105.6	142.0
Mg	212	39.0	62.6
Mn	212	2.6	2.9
Na	212	306.0	301.4
Ni	212	2.3	2.7
P	212	5.4	17.8
Pb	212	1.4	2.6
S	212	401.6	293.3
Se	212	1.6	1.8
Si	212	165.7	203.3
Sr	212	1.8	6.7
Ti	212	10.2	12.7
V	212	0.6	1.2
Zn	212	13.1	17.5

The distributions of home characteristics, indoor PM sources, and selected time activities as reported in questionnaires or derived from GPS are presented in Table S2. Based on the exit survey, 60% of participants spent some time near traffic when outdoors, and 34% spent more than 2 hrs per day commuting during the 48-hr monitoring period. During sampling, 60% of participants opened windows more than half of the time, 26% used air conditioning and 37% used fans at home. In terms of indoor PM sources, 38% were close to cooking smoke, 24% were close to burning candles or incense, and 39% spent time close to people smoking (cigarette, cigar, hookah, or pipe smoke) nearby. In addition, 56% of participants lived in an apartment, 44% were part of a household with >3 persons, and 43% lived in a home built after the 1980s. Participants also spent an estimated mean (SD) of their time at home 78.5% (19.6%) and at non-home locations 15.2% (15.7%).

### Source apportionment results

Fourteen observations had missing values and were replaced by species median, including PM mass (3 obs), BC (3), BrC (6), and ETS (2). A five-factor solution combined fuel oil and secondhand smoking, while seven factors resulted in a non-interpretable factor with single high loading of Zn. A six-factor solution was chosen as the optimal, physically interpretable one (*Q*_robust_ = 5845.4 and *Q*_true_ = 6143.2). An Fpeak rotation of 0.1 was then applied with 100 bootstraps, which resulted in no unmapped factors (Table S4). Some species with higher S/N ratio, e.g., BC, Cl, K, S, Ca, and Zn, had non-normal residuals (Table S5).

**Fig. 1:**
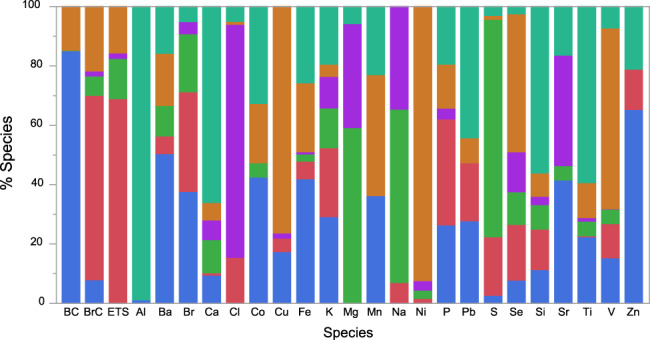
PMF-predicted source loading profiles (in % of species). Sources are color coded as shown: .

The six sources together explained 48% of the variability in PM_2.5_ mass concentration and included the following: Traffic, Secondhand Smoking, Aged Sea Salt, Fresh Sea Salt, Fuel Oil, Crustal (Fig. 1). Each of these is presented in more detail below along with supporting relationships from confirmatory bivariate analyses.

#### Traffic

The first source identified was traffic with high loadings of BC, Zn, and Ba (Fig. 1). It contributed on average 2.6% of personal PM_2.5_ mass (Table 2). Traffic was moderately positively correlated with crustal and inversely correlated with fresh sea salt and fuel oil sources (Table 3). It was strongly correlated with outdoor residential ambient (NO_2_ and PM_2.5_) and traffic-related (total CALINE4 NO_*x*_) air pollutants. It was also negatively correlated with outdoor O_3_. In addition, Traffic source contributions increased with greater length of primary roads in the KDE activity space (Table 4).

**Table 2:** Predicted source contributions to personal PM_2.5_ mass concentrations.

Sources	Average (SD) mass contribution (μg/m^3^)	% contribution to personal PM_2.5_ mass (%)
Traffic	0.5 (0.6)	2.6
Secondhand smoking	11.9 (9.2)	65.3
Aged sea salt	0.8 (0.8)	4.3
Fresh sea salt	0.9 (2.1)	4.7
Fuel oil	2.1 (1.6)	11.7
Crustal	2.1 (3.6)	11.5

**Table 3:**
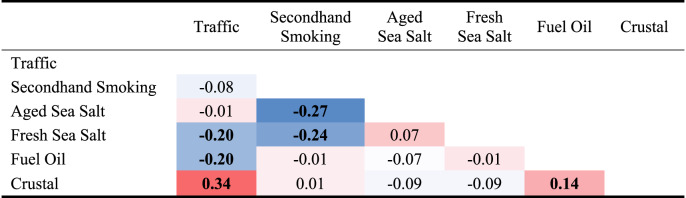
Spearman correlation matrix of PMF-predicted PM_2.5_ source contributions, colored from low (blue) to high (red).

Values in bold font represent significant *p* values at *p* < 0.05 level.

**Table 4:** Spearman correlations between PMF-predicted PM_2.5_ source contributions and select continuous variables.

Sources	Variables	Spearman correlation
Traffic	Outdoor air pollution at the residence	
	O_3_	**−0.35**
	NO_2_	**0.61**
	PM_2.5_	**0.43**
	Total NO_x_ from local traffic on all road classes (CALINE4)	**0.15**
	Total length of primary roads within KDE area	**0.15**
Secondhand Smoking	Ambient air pollution at Downtown Los Angeles monitoring site	
	Potassium ion (K^+^)	0.12
	Potassium (element, K)	−0.04
	Elemental carbon	0.04
	Organic carbon	0.09
Aged Sea Salt	Outdoor air pollution and meteorology at residence	
	O_3_	**0.53**
	Wind speed	**−0.22**
	Temperature	**0.55**
Fresh Sea Salt	Outdoor meteorology at residence	
	Wind speed	**0.27**
	Relative humidity	**0.16**
	Ambient air pollution at Downtown Los Angeles monitoring site	
	Chloride ion (Cl^-^)	**0.24**
	Chlorine (element, Cl)	**0.20**
Fuel Oil	Outdoor air pollution at residence	
	O_3_	**−0.18**
	NO_2_	**0.17**
Crustal	Outdoor air pollution and meteorology at residence	
	NO_2_	**0.39**
	PM_10_	**0.24**
	Relative humidity	**−0.48**
	Precipitation	**−0.16**

Values in bold font represent significant *p* values at *p* < 0.05 level.

#### Secondhand Smoking

**Fig. 2:**
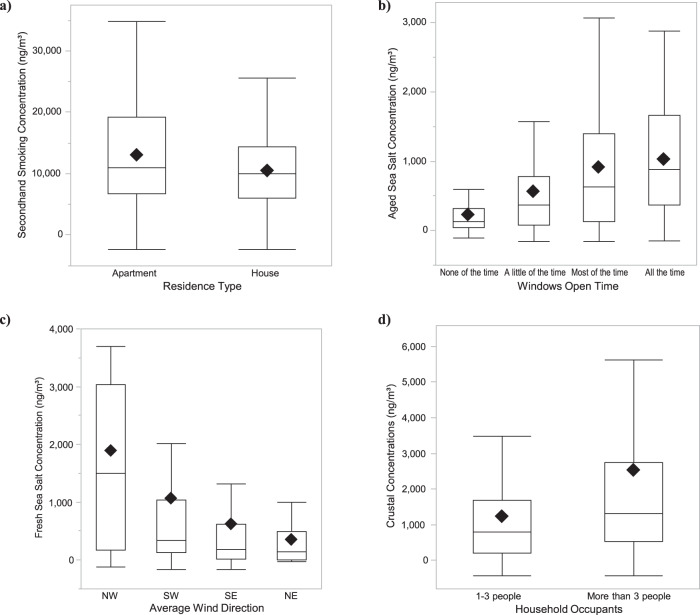
Relationship between source mass contributions (y-axis) and environmental or home characteristics. **a** Secondhand smoking and home type, **b** aged sea salt and window opening time in the 48-h monitoring period, **c** fresh sea salt and average wind direction in the 48-h monitoring period, and **d** crustal and number of household occupants (*The relationships in (**b** **d**) were significant with Kruskal-Wallis test *p* value  < 0.05) .

This second source had a high loading of BrC and ETS (Fig. 1). With an average mass contribution of 11.9 μg/m^3^, it contributed the majority of personal PM_2.5_ mass (65.3% on average) (Table 2). Participants living in apartments seemed to have slightly higher exposure to this source compared to those living in houses (13.0 vs. 10.5 μg/m^3^, respectively, not significant, Fig. 2a). This source was also slightly negatively correlated with greater window opening time. Secondhand smoking (SHS) was also slightly higher when participants were close to 2+ persons smoking nearby as compared to one (among ~33% of participants reporting being close to someone smoking in the 48-hr monitoring period) (13.2 vs. 11.0 μg/m^3^ respectively, not significant). To eliminate the possibility that this source is capturing an outdoor biomass burning signal, we checked its correlation with outdoor potassium (elemental, K), potassium ion (K^+^), and elemental and organic carbon concentrations at the Downtown CSN site, all of which showed insignificant weak correlations (Table 4). SHS was also not correlated with any questionnaire variables on indoor cooking (frequency, use, etc.).

#### Aged sea salt

This third source had high loadings of Na, Mg, and S (Fig. 1). It contributed on average 4.3% of the personal PM_2.5_ mass (Table 2). Aged sea salt was negatively correlated with the SHS source (Table 3). It was strongly positively correlated with outdoor O_3_ concentration and temperature and negatively correlated with wind speed (Table 4). Aged sea salt was also significantly positively correlated with window opening time (Fig. 2b).

#### Fresh sea salt

This fourth source had high loadings of Cl, Na, and Mg (Fig. 1). It contributed on average 4.7% of the personal PM_2.5_ mass (Table 2). Fresh sea salt was negatively correlated with traffic and SHS sources (Table 3). The mass contribution of this source were significantly different across different wind directions, with the highest contribution on days when average wind direction originated from the west (NW followed by SW, Fig. 2c). It was also positively correlated with outdoor residential wind speed and relative humidity. To eliminate the possibility of this being an indoor source related to aerosolized minerals from domestic water use or salt used in cooking [32, 35, 51], we checked its relationships with humidifier usage and time close to smoke from cooking, respectively. Even though the sample size was unbalanced (30 out of 212 reported using a humidifier), average mass contributions were lower (not significant) when people used a humidifier compared to not (0.5 vs. 0.9 μg/m^3^, respectively). Similarly, mass contributions were lower when participants reported spending more time close to smoke from cooking. In addition, fresh sea salt was moderately positively correlated with ambient Cl and Cl^−^ as measured at the Downtown Los Angeles site (Table 4).

#### Fuel oil

This source had high loadings of Cu, Ni, and V (Fig. 1). It contributed the second largest share of personal PM_2.5_ mass (11.7%) with an average mass contribution of 2.1 μg/m^3^ (Table 2). Fuel oil was positively correlated with crustal and negatively correlated with traffic sources (Table 3). This source was slightly higher (non-significant) in participants living in homes built before 1980 compared to after (2.4 vs. 1.9 μg/m^3^). Fuel oil was also positively correlated with outdoor NO_2_ and negatively correlated with O_3_ (Table 4).

#### Crustal

The sixth and final source had high loadings of crustal elements Ca, Si, Ti, and Al (Fig. 1). It contributed 11.5% (2.1 μg/m^3^) of personal PM_2.5_ mass on average (Table 2). Crustal was moderately positively correlated with traffic and fuel oil sources (Table 3). Living in a household with 4+ occupants was associated with greater contributions of this source compared to fewer occupants (2.5 vs. 1.2 μg/m^3^, *p* value = 0.04, Fig. 2d). It was also positively correlated with outdoor NO_2_ and PM_10_ concentrations and negatively correlated with relative humidity and precipitation (Table 4).

## Discussion

We identified six sources of personal PM_2.5_ exposure along with their mass contributions in the 3rd trimester of pregnancy, in a population of 212 low-income, predominantly Hispanic pregnant women living in Los Angeles, CA. Secondhand smoking (SHS) followed by fuel oil and crustal were the biggest contributors to personal PM_2.5_ exposures. Of the six sources, SHS and crustal appeared to be of indoor origin (or closely related to indoor activities), while traffic, aged and fresh sea salt, and fuel oil were of outdoor origin. Secondhand smoke was the single largest contributor to total personal PM_2.5_ mass concentrations. The combined indoor source contributions (14 ± 10 μg/m^3^) were more than triple those of outdoor sources (4.2 ± 2.9 μg/m^3^), highlighting the importance of the indoor environment in contributing to personal exposures during this critical window of time.

SHS is also a well-known indoor air contaminant [52, 53] associated with a suite of adverse health effects [54–56]. This source had high loadings of BrC and ETS (the lab-reported marker of SHS), and some loadings of Br and K which were related to tobacco smoke in previous studies [39, 57, 58]. In order to avoid overloading the samplers with particles from primary tobacco smoke which would also overshadow chemical fingerprints from other sources if present, by design, we excluded participants who reported smoking themselves (this did not occur in this population) or those with an active smoker permanently residing in their household (despite this latter criterion not being consistently applied throughout the study). Despite the low smoking prevalence and these design restrictions, secondhand smoking was still identified as the source with the largest contribution to personal PM_2.5_ exposures in our study. The mass contributions of this source (and measured ETS concentrations) did not show any clear changes over time as the study progressed, suggesting that recruitment decisions did not significantly influence our findings. This source has been found in other personal PM_2.5_ monitoring studies [34, 35, 59]. Minguillón et al. [34] identified cigarette sources in the personal, indoor and outdoor environments for the pregnant women living in Barcelona, Spain. Özkaynak et al. [35] found nicotine mainly in the personal sample and indoor environment for the nonsmoking residents in Riverside, California. Using a real-time PM_2.5_ monitor in personal and residential environment, Zhang et al. [59] found SHS distribution in children with and without self-reported SHS exposure in New York City, where children from smoking families had six times that of SHS exposure for children from non-smoking families. All these studies, including the current one, demonstrate that SHS source is a common finding in the personal monitoring studies. This suggests that secondhand (and possibly thirdhand) smoke exposure remains an issue of public health concern especially during pregnancy and in the context of our population, despite the success of large-scale bans on public smoking. We found that participants living in apartments tended to have marginally higher exposure to secondhand smoking than those living in detached houses. This could suggest greater potential of secondhand smoke infiltration from adjacent units in an apartment building or from visitors smoking [60–62]. Anecdotal evidence from the study (data not shown) also suggests secondhand smoke exposure could be occurring outside of the residence, during public transit or while commuting, and warrants further investigation.

We also found both fresh and aged sea salt—sources of outdoor origin—contributed to personal PM_2.5_ exposures in our study with high loading of Na, Mg, Cl (fresh only), and S (aged only). Previous work identified sea salt or marine aerosol sources with similar loading profiles [16, 23, 63]. Despite only having access to average wind direction data over the 48-hr monitoring period (not an ideal wind measure compared to most frequent wind direction for example), fresh sea salt mass contributions were higher with westerly winds and higher wind speeds, which provides greater potential for aerosolization and airborne transport of sea salt particles from the Pacific Ocean. Habre et al. [23] found sea salt mass contributions to PM_2.5_ mass in southern California to be highest in coastal communities. As fresh sea salt ages and undergoes photochemical reactions that also lead to secondary O_3_ formation, chlorine is lost and sulfates are formed [23, 64]. Thus, aged sea salt resembles fresh sea salt in its loading profiles, except with S substituting Cl. Lower wind speed, stagnant conditions, and warmer temperatures provide more chemical reaction time and contribute to the loss of Cl and formation of O_3_ as fresh sea salt ages [65, 66]. Given the mild, coastal southern California climate year-round, it is not surprising to see both outdoor sea salt sources contributing to personal PM_2.5_ exposures of pregnant women in our study.

As for the crustal source, crustal elements such as Al, Ca, and Ti are naturally present in the earth’s crust. They originate outdoors and can enter the indoor environment as windblown dust or as dust tracked indoors on residents’ shoes. Once indoors, crustal materials will typically settle and get resuspended as indoor sources (or emissions of indoor origin) when disturbed by human movement or other activities (i.e., vacuuming). Therefore, the presence of more occupants in a household provides greater opportunities for re-suspension of crustal dust, similar to earlier studies [26]. Despite crustal being positively correlated with the traffic source, it did not have any loadings of known traffic-related markers (BC, Ba, Zn, Cu) suggesting it was not reflecting a road dust signal [67]. As such, crustal can be thought of as a predominantly indoor origin source despite the possibility of our participants getting exposed to crustal dust outside of their homes, in daily commutes, and outdoor activities.

Finally, we found that fuel oil and traffic sources contributed to personal PM_2.5_ exposures as well. Similar to previous studies, the fuel oil source had high loadings of Ni and V which are known tracers of heavy residual fuel oil combustion in industrial applications, certain heavy-duty machinery, and in marine engine emissions [68–72]. BC serves as a traffic marker especially for diesel engines [16, 26], while Zn, Ba, and Fe are related to motor vehicle exhaust emission, brake and tire wear, and diesel additives [73, 74]. Traffic is an important source of air pollution in Los Angeles, CA [23, 25]. Our study shows that greater concentrations of traffic-related pollutants outdoors (CALINE NO_*x*_ and ambient NO_2_) and spending more time near primary roads (in actual activity spaces) correlate with greater personal exposure to the PM_2.5_ traffic source. It is also important to note that the traffic source had high loadings of tailpipe combustion markers (BC) and non-exhaust brake and tire wear markers (Ba and Zn, respectively) potentially capturing both tailpipe and non-tailpipe traffic exposures.

The strengths of this study include the personal PM_2.5_ measurements and detailed chemical composition analysis that allowed us to apportion the major sources of personal PM_2.5_ exposure during a critical time window of pregnancy. By integrating concurrently collected questionnaire data and geospatially modeled environmental exposures in activity spaces (from GPS) and in the residential neighborhood, the results further corroborate these sources, their determinants, and origin (primarily indoor vs outdoor). However, the PMF model only explained a portion of the variability in personal PM_2.5_ mass concentrations (*R*^2^ 0.48). One possible reason could be that we did not measure organic carbon (OC) species on the Teflon filters in this study which are known to contribute a large fraction of indoor PM_2.5_ mass concentrations [26, 75]. It was also possible that some of most volatile OC species and ammonium nitrate among others volatilized off the filters; we found higher temperature was associated with lower personal PM_2.5_ mass in bivariate analysis [38]. We also did not measure volatile organic compounds or other species that could potentially contribute significantly to personal air pollution exposures (in different phases, and not just in PM_2.5_). The sample size of the study, while considered large in personal monitoring settings, and the short monitoring period may limit the generalizability and representativeness of personal PM_2.5_ exposures beyond this study area and across the full pregnancy and postpartum periods.

## Conclusion

This is one of the few studies to conduct a thorough characterization of sources impacting personal PM_2.5_ exposures of predominantly Hispanic and low-income women during pregnancy in an environmental health disparities context. Our findings show the diversity of sources that impact personal PM_2.5_ exposures in pregnancy in Los Angeles, CA. With ~ 77% of personal exposures contributed by indoor sources, the findings highlight the importance of the indoor environment contributions to total PM_2.5_ exposures during pregnancy and the potentially incomplete understanding of this population’s exposures by solely relying on outdoor air pollution measures. These results may inform source-specific health investigations and subsequent interventions to reduce exposure to potentially more harmful components of PM_2.5_.

## Supplementary information

SUPPLEMENT

## Data Availability

The datasets generated during and/or analyzed during the current study are not publicly available due to human subjects research protections but are available from the corresponding author upon reasonable request.

## References

[1] Larson T, Gould T, Simpson C, Liu LJS, Claiborn C, Lewtas J. Source apportionment of indoor, outdoor, and personal PM_2.5_ in Seattle, Washington, using positive matrix factorization. J Air Waste Manag Assoc. 2004;54:1175–87. 10.1080/10473289.2004.10470976 15468670 10.1080/10473289.2004.10470976

[2] Shang J, Khuzestani RB, Tian J, Schauer JJ, Hua J, Zhang Y, et al. Chemical characterization and source apportionment of PM_2.5_ personal exposure of two cohorts living in urban and suburban Beijing. Environ Pollut. 2019;246:225–36. 10.1016/j.envpol.2018.11.076 30557796 10.1016/j.envpol.2018.11.076

[3] Dadvand P, Ostro B, Amato F, Figueras F, Minguillón MC, Martinez D, et al. Particulate air pollution and preeclampsia: a source-based analysis. Occup Environ Med. 2014;71:570–7. 10.1136/oemed-2013-101693 24683010 10.1136/oemed-2013-101693

[4] Hu H, Ha S, Henderson BH, Warner TD, Roth J, Kan H, et al. Association of atmospheric particulate matter and ozone with gestational diabetes mellitus. Environ Health Perspect. 2015;123:853–9. 10.1289/ehp.1408456 25794412 10.1289/ehp.1408456 PMC4559952

[5] Jedrychowski WA, Perera FP, Maugeri U, Spengler J, Mroz E, Flak E, et al. Prohypertensive effect of gestational personal exposure to fine particulate matter. prospective cohort study in non-smoking and non-obese pregnant women. Cardiovasc Toxicol. 2012;12:216–25. 10.1007/s12012-012-9157-z 22328329 10.1007/s12012-012-9157-z PMC3404286

[6] Guo T, Wang Y, Zhang H, Zhang Y, Zhao J, Wang Q, et al. The association between ambient PM_2.5_ exposure and the risk of preterm birth in China: a retrospective cohort study. Sci Total Environ. 2018;633:1453–9. 10.1016/j.scitotenv.2018.03.328 29758897 10.1016/j.scitotenv.2018.03.328

[7] Percy Z, DeFranco E, Xu F, Hall ES, Haynes EN, Jones D, et al. Trimester specific PM_2.5_ exposure and fetal growth in Ohio, 2007–2010. Environ Res. 2019;171:111–8. 10.1016/j.envres.2019.01.031 30660917 10.1016/j.envres.2019.01.031 PMC6382528

[8] Sun X, Luo X, Zhao C, Zhang B, Tao J, Yang Z, et al. The associations between birth weight and exposure to fine particulate matter (PM_2.5_) and its chemical constituents during pregnancy: a meta-analysis. Environ Pollut. 2016;211:38–47. 10.1016/j.envpol.2015.12.022 26736054 10.1016/j.envpol.2015.12.022

[9] Berger K, Malig BJ, Hasheminassab S, Pearson DL, Sioutas C, Ostro B, et al. Associations of source-apportioned fine particles with cause-specific mortality in California. Epidemiology. 2018;29:639–48. 10.1097/EDE.0000000000000873 29889687 10.1097/EDE.0000000000000873

[10] Habre R, Moshier E, Castro W, Nath A, Grunin A, Rohr A, et al. The effects of PM_2.5_ and its components from indoor and outdoor sources on cough and wheeze symptoms in asthmatic children. J Expo Sci Environ Epidemiol. 2014;24:380–7. 10.1038/jes.2014.21 24714073 10.1038/jes.2014.21

[11] Lee HJ, Park HY. Prioritizing the control of emission sources to mitigate PM_2.5_ disparity in California. Atmos Environ. 2020;224:117316 10.1016/j.atmosenv.2020.117316

[12] Tessum CW, Paolella DA, Chambliss SE, Apte JS, Hill JD, Marshall JD. PM_2.5_ polluters disproportionately and systemically affect people of color in the United States. Sci Adv. 2021;7:1–7. 10.1126/sciadv.abf4491 10.1126/sciadv.abf4491 PMC11426197 33910895

[13] Kim D, Sass-Kortsak A, Purdham JT, Dales RE, Brook JR. Sources of personal exposure to fine particles in Toronto, Ontario, Canada. J Air Waste Manag Assoc. 2005;55:1134–46. 10.1080/10473289.2005.10464710 16187583 10.1080/10473289.2005.10464710

[14] Reff A, Bhave PV, Simon H, Pace TG, Pouliot GA, Mobley JD, et al. Emissions inventory of PM_2.5_ trace elements across the United States. Environ Sci Technol. 2009;43:5790–6. 10.1021/es802930x 19731678 10.1021/es802930x

[15] Watson JG, Chen LWA, Chow JC, Doraiswamy P, Lowenthal DH. Source apportionment: Findings from the U.S. supersites program. J Air Waste Manag Assoc. 2008;58:265–88. 10.3155/1047-3289.58.2.265 18318341 10.3155/1047-3289.58.2.265

[16] Hasheminassab S, Daher N, Ostro BD, Sioutas C. Long-term source apportionment of ambient fine particulate matter (PM_2.5_) in the Los Angeles Basin: a focus on emissions reduction from vehicular sources. Environ Pollut. 2014;193:54–64. 10.1016/j.envpol.2014.06.012 25005887 10.1016/j.envpol.2014.06.012

[17] Hopke PK. Recent developments in receptor modeling. J Chemometr. 2003;17:255–65. 10.1002/cem.796

[18] Hopke PK. Review of receptor modeling methods for source apportionment. J Air Waste Manag Assoc. 2016;66:237–59. 10.1080/10962247.2016.1140693 26756961 10.1080/10962247.2016.1140693

[19] Norris G, Duvall R, Brown S, Bai S. EPA positive matrix factorization (PMF) 5.0 fundamentals and user guide. (Environmental Protection Agency Office of Research and Development; Publishing House, Washington, DC, 2014).

[20] Paatero P, Tapper U. Positive matrix factorization: a non‐negative factor model with optimal utilization of error estimates of data values. Environmetrics. 1994;5:111–26.

[21] Bell ML, Belanger K, Ebisu K, Gent JF, Lee HJ, Koutrakis P, et al. Parental exposure to fine particulate matter and birth weight: variations by particulate constituents and sources. Epidemiology. 2010;21:884–91. 10.1097/EDE.0b013e3181f2f405.Prenatal 20811286 10.1097/EDE.0b013e3181f2f405 PMC3055585

[22] Rohr AC, Habre R, Godbold J, Moshier E, Schachter N, Kattan M, et al. Asthma exacerbation is associated with particulate matter source factors in children in New York City. Air Qual Atmos Health. 2014;7:239–50. 10.1007/s11869-013-0230-y

[23] Habre R, Girguis M, Urman R, Fruin S, Lurmann F, Shafer M, et al. Contribution of tailpipe and non-tailpipe traffic sources to quasi-ultrafine, fine and coarse particulate matter in southern California. J Air Waste Manag Assoc. 2021;71:209–30. 10.1080/10962247.2020.1826366 32990509 10.1080/10962247.2020.1826366 PMC8112073

[24] Hasheminassab S, Daher N, Schauer JJ, Sioutas C. Source apportionment and organic compound characterization of ambient ultrafine particulate matter (PM) in the Los Angeles Basin. Atmos Environ. 2013;79:529–39. 10.1016/j.atmosenv.2013.07.040

[25] Hasheminassab S, Daher N, Shafer MM, Schauer JJ, Delfino RJ, Sioutas C. Chemical characterization and source apportionment of indoor and outdoor fine particulate matter (PM_2.5_) in retirement communities of the Los Angeles Basin. Sci Total Environ. 2014;490:528–37. 10.1016/j.scitotenv.2014.05.044 24880542 10.1016/j.scitotenv.2014.05.044 PMC4098872

[26] Habre R, Coull B, Moshier E, Godbold J, Grunin A, Nath A, et al. Sources of indoor air pollution in New York City residences of asthmatic children. J Expo Sci Environ Epidemiol. 2014;24:269–78. 10.1038/jes.2013.74 24169876 10.1038/jes.2013.74

[27] MacIntosh DL, Spengler JD, World Health Organization. Human exposure assessment. World Health Organization, 2000. Retrieved from https://apps.who.int/iris/handle/10665/42181

[28] Ott WR, Steinemann AC, Wallace LA. Exposure analysis (CRC Press 2006).

[29] Jedrychowski W, Perera F, Mrozek-Budzyn D, Mroz E, Flak E, Spengler JD, et al. Gender differences in fetal growth of newborns exposed prenatally to airborne fine particulate matter. Environ Res. 2009;109:447–56. 10.1016/j.envres.2009.01.009 19261271 10.1016/j.envres.2009.01.009 PMC3786262

[30] Rundle A, Hoepner L, Hassoun A, Oberfield S, Freyer G, Holmes D, et al. Association of childhood obesity with maternal exposure to ambient air polycyclic aromatic hydrocarbons during pregnancy. Am J Epidemiol. 2012;175:1163–72. 10.1093/aje/kwr455 22505764 10.1093/aje/kwr455 PMC3491973

[31] Jenkins PL, Phillips TJ, Mulberg EJ, Hui SP. Activity patterns of Californians: use of and proximity to indoor pollutant sources. Atmos Environ Part A Gen Top. 1992;26:2141–8. 10.1016/0960-1686(92)90402-7

[32] Wallace L. Indoor particles: a review. J Air Waste Manag Assoc. 1996;46:98–126. 10.1080/10473289.1996.10467451 8846246 10.1080/10473289.1996.10467451

[33] Brinkman GL, Milford JB, Schauer JJ, Shafer MM, Hannigan MP. Source identification of personal exposure to fine particulate matter using organic tracers. Atmos Environ. 2009;43:1972–81. 10.1016/j.atmosenv.2009.01.023

[34] Minguillón MC, Schembari A, Triguero-Mas M, de Nazelle A, Dadvand P, Figueras F, et al. Source apportionment of indoor, outdoor and personal PM_2.5_ exposure of pregnant women in Barcelona, Spain. Atmos Environ. 2012;59:426–36. 10.1016/j.atmosenv.2012.04.052

[35] Özkaynak H, Xue J, Spengler J, Wallace L, Pellizzari E, Jenkins P. Personal exposure to airborne particles and metals: results from the particle TEAM study in Riverside, California. J Expo Anal Environ Epidemiol. 1996;6:57–78. 8777374

[36] Bastain TM, Chavez T, Habre R, Girguis MS, Grubbs B, Toledo-Corral C, et al. Study design, protocol and profile of the maternal and developmental risks from environmental and social stressors (MADRES) pregnancy cohort: a prospective cohort study in predominantly low-income Hispanic women in urban Los Angeles. BMC Preg Childbirth. 2019;19:1–16. 10.1186/s12884-019-2330-7 10.1186/s12884-019-2330-7 PMC6543670 31146718

[37] O’Sharkey K, Xu Y, Chavez T, Johnson M, Cabison J, Rosales M, et al. In-utero personal exposure to PM_2.5_ impacted by indoor and outdoor sources in the MADRES cohort. Environ Adv. 2022;9:100257 10.2139/ssrn.4076820 36778968 10.1016/j.envadv.2022.100257 PMC9912940

[38] Xu Y, Yi L, Cabison J, Rosales M, O’Sharkey K, Chavez TA, et al. The impact of GPS-derived activity spaces on personal PM_2.5_ exposures in the MADRES cohort. *Environ Res.* 2022;214. 10.1016/j.envres.2022.114029 10.1016/j.envres.2022.114029 PMC11905758 35932832

[39] Lawless PA, Rodes CE, Ensor DS. Multiwavelength absorbance of filter deposits for determination of environmental tobacco smoke and black carbon. Atmos Environ. 2004;38:3373–83. 10.1016/j.atmosenv.2004.03.038

[40] Yan B, Kennedy D, Miller RL, Cowin JP, Jung K, Perzanowski M, et al. Validating a nondestructive optical method for apportioning colored particulate matter into black carbon and additional components. Atmos Environ. 2011;45:7478–86. 10.1016/j.atmosenv.2011.01.044 10.1016/j.atmosenv.2011.01.044 PMC3223915 22125411

[41] Benson PE. A review of the development and application of the CALINE3 and 4 models. Atmos Environ Part B Urban Atmos. 1992;26:379–90. 10.1016/0957-1272(92)90013-I

[42] Abatzoglou JT. Development of gridded surface meteorological data for ecological applications and modelling. Int J Climatol. 2013;33:121–31. 10.1002/joc.3413

[43] Cich G, Knapen L, Bellemans T, Janssens D, Wets G. Threshold settings for TRIP/STOP detection in GPS traces. J Ambient Intell Hum Comput. 2016;7:395–413. 10.1007/s12652-016-0360-9

[44] Pérez-Torres R, Torres-Huitzil C, Galeana-Zapién H. Full on-device stay points detection in smartphones for location-based mobile applications. *Sensors*. 2016;16. 10.3390/s16101693 10.3390/s16101693 PMC5087481 27754388

[45] Jankowska MM, Schipperijn J, Kerr J. A framework for using GPS data in physical activity and sedentary behavior studies. Exerc Sport Sci Rev. 2015;43:48. 25390297 10.1249/JES.0000000000000035 PMC4272622

[46] Sherman JE, Spencer J, Preisser JS, Gesler WM, Arcury TA. A suite of methods for representing activity space in a healthcare accessibility study. Int J Health Geogr. 2005;4:1–21. 10.1186/1476-072X-4-24 16236174 10.1186/1476-072X-4-24 PMC1283149

[47] Jankowska MM, Natarajan L, Godbole S, Meseck K, Sears DD, Patterson RE, et al. Kernel density estimation as a measure of environmental exposure related to insulin resistance in breast cancer survivors. Cancer Epidemiol Biomark Prev. 2017;26:1078–84. 10.1158/1055-9965.EPI-16-0927 10.1158/1055-9965.EPI-16-0927 PMC5500421 28258052

[48] Solomon PA, Crumpler D, Flanagan JB, Jayanty RKM, Rickman EE, McDade CE. U.S. National PM_2.5_ chemical speciation monitoring networks - CSN and IMPROVE: description of networks. J Air Waste Manag Assoc. 2014;64:1410–38. 10.1080/10962247.2014.956904 25562937 10.1080/10962247.2014.956904

[49] Paatero P. Least squares formulation of robust non-negative factor analysis. Chemometr Intell Lab Syst. 1997;37:23–35. 10.1016/S0169-7439(96)00044-5

[50] Brown SG, Frankel A, Hafner HR. Source apportionment of VOCs in the Los Angeles area using positive matrix factorization. Atmos Environ. 2007;41:227–37. 10.1016/j.atmosenv.2006.08.021

[51] Schachter EN, Rohr A, Habre R, Koutrakis P, Moshier E, Nath A, et al. Indoor air pollution and respiratory health effects in inner-city children with moderate to severe asthma. Air Qual Atmos Health. 2020;13:247–57. Retrieved from 10.1007/s11869-019-00789-3

[52] Mueller D, Uibel S, Braun M, Klingelhoefer D, Takemura M, Groneberg DA. Tobacco smoke particles and indoor air quality (ToPIQ)—the protocol of a new study. J Occup Med Toxicol. 2011;6:1–5. 10.1186/1745-6673-6-35 22188808 10.1186/1745-6673-6-35 PMC3260229

[53] Nazaroff WW, Singer BC. Inhalation of hazardous air pollutants from environmental tobacco smoke in US residences. *J Expo Anal Environ Epidemiol.* 2004;14. 10.1038/sj.jea.7500361 10.1038/sj.jea.7500361 15118748

[54] Chen R, Clifford A, Lang L, Anstey KJ. Is exposure to secondhand smoke associated with cognitive parameters of children and adolescents? A systematic literature review. Ann Epidemiol. 2013;23:652–61. 10.1016/j.annepidem.2013.07.001 23969303 10.1016/j.annepidem.2013.07.001

[55] Flouris AD, Vardavas CI, Metsios GS, Tsatsakis AM, Koutedakis Y. Biological evidence for the acute health effects of secondhand smoke exposure. *Am J Physiol*— *Lung Cell Mol Physiol.* 2010;298. 10.1152/ajplung.00215.2009 10.1152/ajplung.00215.2009 19767410

[56] Leonardi-Bee J, Britton J, Venn A. Secondhand smoke and adverse fetal outcomes in nonsmoking pregnant women: a meta-analysis. Pediatrics. 2011;127:734–41. 10.1542/peds.2010-3041 21382949 10.1542/peds.2010-3041

[57] Benner CL, Bayona JM, Caka FM, Tang H, Lewis L, Crawford J, et al. Chemical composition of environmental tobacco smoke. 2. particulate-phase compounds. Environ Sci Technol. 1989;23:688–99. 10.1021/es00064a007

[58] Müller ALH, Bizzi CA, Pereira JSF, Mesko MF, Moraes DP, Floresa EMM, et al. Bromine and chlorine determination in cigarette tobacco using microwave-induced combustion and inductively coupled plasma optical emission spectrometry. J Braz Chem Soc. 2011;22:1649–55. 10.1590/s0103-50532011000900005

[59] Zhang T, Chillrud SN, Yang Q, Pitiranggon M, Ross J, Perera F, et al. Characterizing peak exposure of secondhand smoke using a real-time PM_2.5_ monitor. Indoor Air. 2020;30:98–107. 10.1111/ina.12611 31610044 10.1111/ina.12611 PMC7137634

[60] Fabian MP, Lee SK, Underhill LJ, Vermeer K, Adamkiewicz G, Levy JI. Modeling environmental tobacco smoke (ETS) infiltration in low-income multifamily housing before and after building energy retrofits. Int J Environ Res Public Health. 2016;13:1–15. 10.3390/ijerph13030327 10.3390/ijerph13030327 PMC4808990 26999174

[61] Price PN, Shehabi A, Chan R. Indoor-outdoor air exchange rates of California apartments and commercial buildings. PIER.LBNL-60682 (California Energy Commission, 2006).

[62] Wilson KM, Klein JD, Blumkin AK, Gottlieb M, Winickoff P, Wilson AKM, et al. Tobacco-smoke exposure in children who live in multiunit housing. Pediatrics. 2011;127:85–92. 10.1542/peds.2010-2046 21149434 10.1542/peds.2010-2046

[63] Corral AF, Dadashazar H, Stahl C, Edwards E, Zuidema P, Sorooshian A. Source apportionment of aerosol at a coastal site and relationships with precipitation chemistry: a case study over the southeast United States. *Atmosphere*. 2020 1212. Retrieved from 10.3390/atmos11111212 10.3390/atmos11111212 PMC8243544 34211764

[64] Gard EE, Kleeman MJ, Gross DS, Hughes LS, Allen JO, Morrical BD, et al. Direct observation of heterogeneous chemistry in the atmosphere. Science. 1998;279:1184–7. 10.1126/science.279.5354.1184 9469803 10.1126/science.279.5354.1184

[65] Crawford J, Cohen DD, Chambers SD, Williams AG, Atanacio A. Impact of aerosols of sea salt origin in a coastal basin: Sydney, Australia. Atmos Environ. 2019;207:52–62.

[66] Knipping EM, Dabdub D. Impact of chlorine emissions from sea-salt aerosol on coastal urban ozone. Environ Sci Technol. 2003;37:275–84. 12564898 10.1021/es025793z

[67] Lough GC, Schauer JJ, Park JS, Shafer MM, Deminter JT, Weinstein JP. Emissions of metals associated with motor vehicle roadways. Environ Sci Technol. 2005;39:826–36. 10.1021/es048715f 15757346 10.1021/es048715f

[68] Corbin JC, Mensah AA, Pieber SM, Orasche J, Michalke B, Zanatta M, et al. Trace metals in soot and PM_2.5_ from heavy-fuel-oil combustion in a marine engine. Environ Sci Technol. 2018;52:6714–22. 10.1021/acs.est.8b01764 29688717 10.1021/acs.est.8b01764 PMC5990929

[69] Manno E, Varrica D, Dongarrà G. Metal distribution in road dust samples collected in an urban area close to a petrochemical plant at Gela, Sicily. Atmos Environ. 2006;40:5929–41. 10.1016/j.atmosenv.2006.05.020

[70] Maykut NN, Lewtas J, Kim E, Larson TV. Source apportionment of PM_2.5_ at an urban IMPROVE site in Seattle, Washington. Environ Sci Technol. 2003;37:5135–42. 10.1021/es030370y 14655699 10.1021/es030370y

[71] Spada NJ, Cheng X, White WH, Hyslop NP. Decreasing vanadium footprint of bunker fuel emissions. Environ Sci Technol. 2018;52:11528–34. 10.1021/acs.est.8b02942 30203968 10.1021/acs.est.8b02942

[72] Zhang Z, Chau PYK, Lai HK, Wong CM. A review of effects of particulate matter-associated nickel and vanadium species on cardiovascular and respiratory systems. Int J Environ Health Res. 2009;19:175–85. 10.1080/09603120802460392 20183191 10.1080/09603120802460392

[73] Ålander T, Antikainen E, Raunemaa T, Elonen E, Rautiola A, Torkkell K. Particle emissions from a small two-stroke engine: effects of fuel, lubricating oil, and exhaust aftertreatment on particle characteristics. Aerosol Sci Technol. 2005;39:151–61. 10.1080/027868290910224

[74] Onat B, Sahin UA, Akyuz T. Elemental characterization of PM_2.5_ and PM_1_ in dense traffic area in Istanbul, Turkey. Atmos Pollut Res. 2013;4:101–5. 10.5094/APR.2013.010

[75] Turpin BJ, Weisel CP, Morandi M, Colome S, Stock T, Eisenreich S, et al. Relationships of indoor, outdoor, and personal air (RIOPA): part II. analyses of concentrations of particulate matter species. Research Report. 1–77 (Health Effects Institute, 2007). 18064946

