# Supplementary material for: Sources of personal PM2.5 exposure during pregnancy in the MADRES cohort
Source: J Expo Sci Environ Epidemiol. 2024 Feb 7;34(5):868–77. doi: 10.1038/s41370-024-00648-z (PMC11446843; doi:10.1038/s41370-024-00648-z)
Supplement: Supplementary file 1 — SUPPLEMENT [file 41370_2024_648_MOESM1_ESM.docx]

**SUPPLEMENT**

**Sources of Personal PM_2.5_ Exposure During Pregnancy in the MADRES Cohort**

Yan Xu^1,2^, Karl O’Sharkey^2^, Jane Cabison^2^, Marisela Rosales^2^, Thomas Chavez^2^, Mark Johnson^2^, Tingyu Yang^2^, Seung-Hyun Cho^3^, Ryan Chartier^3^, Brendan Grubbs^4^, Nathana Lurvey^5^, Deborah Lerner^5^, Frederick Lurmann^6^, Shohreh Farzan^2^, Theresa Bastain^2^, Carrie Breton^2^, John P. Wilson^1,2,7^, Rima Habre^1,2^

^1^Spatial Sciences Institute, University of Southern California; ^2^Department of Population and Public Health Sciences, University of Southern California; ^3^Research Triangle Institute International, Inc., Research Triangle Park, North Carolina; ^4^Department of Obstetrics and Gynecology, University of Southern California; ^5^Eisner Health, Los Angeles, California; ^6^Sonoma Technology, Inc., Petaluma, California; ^7^Department of Civil & Environmental Engineering, Computer Science, and Sociology, University of Southern California

**FUNDING**

The study was supported by NIEHS R01ES027409, NIEHS P30ES007048 pilot funding, the MADRES Center (NIEHS/NIMHD P50ES026086, EPA 83615801, NIMHD P50MD015705), and fellowships (1^st^ and 5^th^ year of study) from the USC Spatial Sciences Institute.

**TABLE OF CONTENTS**

[Table S1: Descriptive statistics of participant demographics (N=212). 2](#_Toc147315548)

[Table S2: Home characteristics, indoor sources, and time-activities derived from questionnaires and exit survey (N=212). 3](#_Toc147315549)

[Table S3: Summary of geospatial data sources obtained from open-sources. 5](#_Toc147315550)

[Table S4: Bootstrapping results for base solution, final rotated Fpeak solution and model variability/error diagnostics. 6](#_Toc147315551)

[Table S5: PMF model results (final rotated solution) showing R^2^ and normality of residuals for each species. 7](#_Toc147315552)

# Table S1: Descriptive statistics of participant demographics (N=212).

| **Variable** | **Mean ± SD or n (%)** | **Variable** | **Mean ± SD or n (%)** |  |
| --- | --- | --- | --- | --- |
| Maternal Age (years) | 28.3 (6.0) | **Employment** |  |  |
| Parity | 2（1.2） | Homemaker | 57 (26.9%) |  |
| **Race** |  | Student | 21 (9.9%) |  |
| White, non-Hispanic | 12 (5.7%) | Employed | 84 (39.6%) |  |
| Asian, non-Hispanic | 2 (0.9%) | Temporary Medical Leave | 9 (4.2%) |  |
| African American, non-Hispanic | 23 (10.8%) | Unemployed | 35 (16.5%) |  |
| Hispanic | 166 (78.3%) | Missing | 6 (2.8%) |  |
| Other | 4 (1.9%) | **Working Status** |  |  |
| Missing | 5 (2.4%) | No | 106 (50.0%) |  |
| **Education** |  | Yes | 101 (47.6%) |  |
| < 12th grade | 50 (23.6%) | Missing | 5 (2.4%) |  |
| Completed high school | 66 (31.1%) | **Household income in the last year** | |  |
| Some college | 59 (27.8%) | Less than $15,000 | 44 (20.7%) |  |
| Completed college | 25 (11.8%) | $15,000 to $29,999 | 47 (22.1%) |  |
| Some Gradate school | 7 (3.3%) | $30,000 to $49,999 | 29 (13.6%) |  |
| Missing | 5 (2.4%) | $50,000 to $99,999 | 7 (3.3%) |  |
|  |  | $100,000 or more | 8 (3.8%) |  |
|  |  | Don't know | 76 (35.7%) |  |
|  |  | Missing | 2 (0.9%) |  |

# Table S2: Home characteristics, indoor sources, and time-activities derived from questionnaires and exit survey (N=212).

| **Variables** | n (%) | **Variables** | n (%) |
| --- | --- | --- | --- |
| **Home Characteristics** |  | **How open were your windows or doors in general? | |
| *Which best describes the home in which you currently live most of the time? | | A little to half way | 86 (40.6%) |
| House | 75 (35.4%) | Most to all the way | 92 (43.4%) |
| Apartment | 118 (55.7%) | Missing | 34 (16.0%) |
| Missing | 19 (9.0%) | **How much of the time was a portable or ceiling fan used in your home, when you were there with the sampler? | |
| *How many people counting yourself live in your household? | | None of the time | 129 (60.8%) |
| 1 and 2 people | 26 (12.3%) | A little, most, or all of the time | 78 (36.8%) |
| 3 people | 29 (13.7%) | Missing | 5 (2.4%) |
| 4 people | 40 (18.9%) | **Indoor Air Pollution Source** |  |
| 5 people | 20 (9.4%) | **How much of the time were you close to smoke from candles or incense burning nearby? | |
| More than 5 people | 34 (15.9%) | None of the time | 158 (74.5%) |
| Missing | 63 (29.7%) | A little, most, or all of the time | 51 (24.1%) |
| *About when was this home building originally built? | | Missing | 3 (1.4%) |
| Built after 1980s | 90 (42.5%) | **How much of the time were you close to smoke or fume from cooking? | |
| Built before 1980s | 68 (32.1%) | None of the time | 129 (60.8%) |
| Missing | 54 (25.5%) | A little, most, or all of the time | 80 (37.7%) |
| *Is there carpeting in your home? |  | Missing | 3 (1.4%) |
| No | 103 (48.6%) | **How much of the time were you close to cigarette, cigar, hookah or pipe smoke from people smoking nearby? | |
| Yes | 91 (42.9%) | None of the time | 125 (59.0%) |
| Missing | 18 (8.5%) | A little, most, or all of the time | 83 (39.2%) |
| *Do you have pets at home? |  | Missing | 4 (1.9%) |
| No | 134 (63.2%) | **Time-Activities** |  |
| Yes | 74 (34.9%) | **How much of the time did you spend outdoors (not commuting in a car, bus or train)? | |
| Missing | 4 (1.9%) | None or a little of the time | 133 (62.7%) |
| *Does your home have heating? |  | Most or all of the time | 76 (35.8%) |
| No | 73 (34.4%) | Missing | 3 (1.4%) |
| Yes | 120 (56.6%) | **When outdoor, whether were you near traffic? | |
| Missing | 19 (9.0%) | No | 81 (38.2%) |
| **Home Ventilation** |  | Yes | 128 (60.4%) |
| ** How long the window open in your home, when you were there with sampler? | | Missing | 3 (1.4%) |
| None or little of the time | 82 (38.7%) | **How many hours did you spend on commute? | |
| Most or all of the time | 127 (59.9%) | 0 to 30 min | 17 (8.0%) |
| Missing | 3 (1.4%) | 30 min to 1 hr | 44 (20.8%) |
| **How much of the time was the air conditioner used in your home, when you were there with the sampler? | | 1 to 2 hrs | 47 (22.2%) |
| None of the time | 154 (72.6%) | > 2 hrs | 72 (34.0%) |
| A little, most, or all of the time | 55 (25.9%) | Missing | 32 (15.1%) |
| Missing | 3 (1.4%) |  |  |

* From the 3^rd^ trimester questionnaire; ** Reported or derived from exit survey referring to 48-hour monitoring period.

# Table S3: Summary of geospatial data sources obtained from open-sources.

| **Geospatial Data** | **Year** | **Web Sources** |
| --- | --- | --- |
| Parks and open space area | 2018 | <https://data.lacounty.gov/Sustainability/LA-County-Parks-and-Open-Space-2018/98vt-tkkj> |
| Normalized Difference Vegetation Index (NDVI) with 0.6 m resolution | 2018 | <https://datagateway.nrcs.usda.gov/GDGHome_DirectDownLoad.aspx> |
| TIGER road network | 2018 | <https://www.census.gov/cgi-bin/geo/shapefiles/index.php?year=2018&layergroup=Roads> |
| TIGER Road Class Codes | 2018 | <https://www2.census.gov/geo/pdfs/reference/mtfccs2018.pdf> |
| Primary road traffic volumes | 2017 | <https://data.ca.gov/dataset/annual-average-daily-traffic-volumes> |
| Walkability index scores at Census 2010 block group level | 2015 | <https://catalog.data.gov/dataset/walkability-index> |

# Table S4: Bootstrapping results for base solution, final rotated Fpeak solution and model variability/error diagnostics.

| Legend |  |
| --- | --- |
| Factor 1 | Traffic |
| Factor 2 | Secondhand smoking |
| Factor 3 | Aged sea salt |
| Factor 4 | Fresh sea salt |
| Factor 5 | Fuel oil |
| Factor 6 | Crustal |

| **Mapping of bootstrap factors to base factors (BS mapping, 100 bootstraps, 0.6 minimum correlation)** | | | | | | | |
| --- | --- | --- | --- | --- | --- | --- | --- |
|  | Factor 1 | Factor 2 | Factor 3 | Factor 4 | Factor 5 | Factor 6 | Unmapped |
|  | 100 | 0 | 0 | 0 | 0 | 0 | 0 |
|  | 4 | 73 | 13 | 6 | 0 | 2 | 2 |
|  | 0 | 0 | 98 | 2 | 0 | 0 | 0 |
|  | 0 | 0 | 0 | 100 | 0 | 0 | 0 |
|  | 0 | 0 | 0 | 0 | 100 | 0 | 0 |
|  | 0 | 0 | 0 | 0 | 0 | 100 | 0 |
| **Mapping of Fpeak (rotated) bootstrap factors to base factors** | | | | | | | |
|  | Base Factor 1 | Base Factor 2 | Base Factor 3 | Base Factor 4 | Base Factor 5 | Base Factor 6 | Unmapped |
| Boot Factor 1 | 100 | 0 | 0 | 0 | 0 | 0 | 0 |
| Boot Factor 2 | 6 | 93 | 1 | 0 | 0 | 0 | 0 |
| Boot Factor 3 | 0 | 0 | 100 | 0 | 0 | 0 | 0 |
| Boot Factor 4 | 0 | 0 | 0 | 100 | 0 | 0 | 0 |
| Boot Factor 5 | 0 | 0 | 0 | 0 | 100 | 0 | 0 |
| Boot Factor 6 | 0 | 0 | 0 | 0 | 0 | 100 | 0 |
| **DISP Diagnostics** | | | | | | | |
| Error Code: | 0 |  |  |  |  |  |  |
| Largest Decrease in Q: | 0 |  |  |  |  |  |  |
| %dQ: | 0 |  |  |  |  |  |  |
| Swaps by Factor: | 0 | 0 | 0 | 0 | 0 | 0 | 0 |
| **BS-DISP Diagnostics** |  |  |  |  |  |  |  |
| BS-DISP Displaced Species: | BrC |  |  |  |  |  |  |
| # of Cases Accepted: | 97 |  |  |  |  |  |  |
| % of Cases Accepted: | 97% |  |  |  |  |  |  |
| Largest Decrease in Q: | -20.35 |  |  |  |  |  |  |
| %dQ: | -0.35 |  |  |  |  |  |  |
| # of Decreases in Q: | 2 |  |  |  |  |  |  |
| # of Swaps in Best Fit: | 0 |  |  |  |  |  |  |
| # of Swaps in DISP: | 1 |  |  |  |  |  |  |
| Swaps by Factor: | 0 | 0 | 0 | 0 | 0 | 0 |  |

# Table S5: PMF model results (final rotated solution) showing R^2^ and normality of residuals for each species.

| Species | R^2^ | Normal residuals? |
| --- | --- | --- |
| PM mass | 0.48 | Yes |
| Carbon Species | |  |
| BC | 0.16 | No |
| BrC | 0.53 | Yes |
| ETS | 0.12 | No |
| Elements |  |  |
| Al | 0.5 | No |
| Ba | 0.41 | Yes |
| Br | 0.24 | Yes |
| Ca | 0.53 | No |
| Cl | 0.85 | No |
| Co | 0.33 | No |
| Cu | 0.77 | Yes |
| Fe | 0.78 | Yes |
| K | 0.13 | No |
| Mg | 0.84 | Yes |
| Mn | 0.54 | Yes |
| Na | 0.86 | Yes |
| Ni | 0.35 | Yes |
| P | 0.0001 | No |
| Pb | 0.13 | No |
| S | 0.83 | No |
| Se | 0.09 | Yes |
| Si | 0.62 | Yes |
| Sr | 0.04 | No |
| Ti | 0.7 | Yes |
| V | 0.04 | No |
| Zn | 0.3 | No |
